# New Insight into the Pathogenesis of Erythema Nodosum Leprosum: The Role of Activated Memory T-Cells

**DOI:** 10.3389/fimmu.2017.01149

**Published:** 2017-09-15

**Authors:** Edessa Negera, Kidist Bobosha, Stephen L. Walker, Birtukan Endale, Rawleigh Howe, Abraham Aseffa, Hazel M. Dockrell, Diana N. Lockwood

**Affiliations:** ^1^London School of Hygiene and Tropical Medicine, Faculty of Infectious Diseases, London, United Kingdom; ^2^Armauer Hansen Research Institute, Addis Ababa, Ethiopia

**Keywords:** erythema nodosum leprosum, ethiopia, leprosy, memory T-cells, prednisolone, reaction

## Abstract

Memory T-cells, particularly, effector memory T cells are implicated in the pathogenesis of inflammatory diseases and may contribute to tissue injury and disease progression. Although erythema nodosum leprosum (ENL) is an inflammatory complication of leprosy, the role of memory T cell subsets has never been studied in this patient group. The aim of this study was at investigate the kinetics of memory T cell subsets in patients with ENL before and after prednisolone treatment. A case–control study design was used to recruit 35 untreated patients with ENL and 25 non-reactional lepromatous leprosy (LL) patient controls at ALERT Hospital, Ethiopia. Venous blood samples were obtained before, during, and after treatment from each patient. Peripheral blood mononuclear cells (PBMCs) were isolated and used for immunophenotyping of T cell activation and memory T-cell subsets by flow cytometry. The kinetics of these immune cells in patients with ENL before and after treatment were compared with LL patient controls as well as within ENL cases at different time points. The median percentage of CD3^+^, CD4^+^, and CD8^+^ T-cells expressing activated T-cells were significantly higher in the PBMCs from patients with ENL than from LL patient controls before treatment. The median percentage of central and activated memory T-cells was significantly increased in patients with ENL compared to LL patient controls before treatment. Interestingly, patients with ENL had a lower percentage of naïve T cells (27.7%) compared to LL patient controls (59.5%) (*P* < 0.0001) before treatment. However, after prednisolone treatment, patients with ENL had a higher median percentage of naïve T-cells (43.0%) than LL controls (33.0%) (*P* < 0.001). The median percentage of activated T-cells (effector memory and effector T-cells) was significantly increased in patients with ENL (59.2%) before treatment compared to after treatment with prednisolone (33.9%) (*P* < 0.005). This is the first work which has shown T-cell activation and the different subsets of memory T cells in untreated patients with ENL. Consequently, this study delineates the role of T-cell activation in the pathogenesis of ENL reaction and challenges the long-standing dogma of immune complex as a sole etiology of ENL reaction.

## Introduction

Leprosy is a disease caused by *Mycobacterium leprae*, an intracellular acid-fast bacillus. It mainly infects the skin and peripheral nerves. Leprosy is a disease with a five-district forms called spectrum with the localized tuberculoid leprosy (TT) and the generalized lepromatous leprosy (LL) forming the two poles of the spectrum (1).

Leprosy reactions [reversal reactions and erythema nodosum leprosum (ENL)] are immune-mediated inflammatory complications of the disease which can occur before, during, or after successful completion of multidrug treatment (2). They cause a significant morbidity and nerve damage in leprosy patients (3). ENL is an inflammatory complication of leprosy, manifesting as tender erythematous skin lesions and systemic features of disease including fever, neuritis, and bone pain (4).

Immunological memory is a characteristic of adaptive immunity. After infection, some antigen experienced T-cells generate memory T-cells which can provide life-long protection against the same infection. These memory T cells rapidly undergo clonal expansion to fight off the reoccurrence of the same infection (5). Memory is a signature of the acquired immune system. It results from antigen-specific lymphocytes clonal expansion and differentiation, which eventually persist for a lifetime (6). Memory lymphocytes ensure immediate protection in peripheral tissues and provide recall responses to antigens in secondary lymphoid organs. In the cellular immune system, these functions are carried out by distinct cell types called effector and central memory T-cells (T_CM_ cells). Protective memory is mediated by effector memory T-cells (T_EM_ cells) that roam to inflamed peripheral tissues and confer immediate effector function, whereas reactive memory is carried out by T_CM_ cells that home to T-cell areas of secondary lymphoid organs (7). T_CM_ cells proliferate and differentiate to effector cells in response to antigenic stimulation (5).

Memory T-cells are characterized into two groups based on their phenotypic and functional profiles as T_CM_ and T_EM_ cells (7). The presence or absence of lymph node homing receptors CD62L (L-selectin) and C–C chemokine receptpr-7 (CCR7) on the cell surface are used to distinguish between T_CM_ and T_EM_ cells (8). CD62L or L-selection is a glycoprotein adhesion molecule which serves as a homing receptor for lymphocytes to enter secondary lymphoid tissues through high endothelial venules. CD62L shed from the surface during T-cell activation resulting in CD62L-negative effector cells (effector cells and T_EM_ cells). Hence, effector memory T-lymphocytes do not express L-selectin, as they circulate in the periphery and have immediate effector functions upon encountering antigen. Unlike T_EM_ cells, CD62L and CCR7 are present on the surface of T_CM_ (7). Naïve (N_TC_) and T_CM_ cells express CD62L and CCR7 for migration to secondary lymphoid organ. In the absence of CD62L and CCR7 molecules, T_EM_ and effector T cells (T_EC_) build up in the peripheral tissues. T_EC_ cells are terminally differentiated memory T-cells. They are the relatively short-lived activated cells whose functions involve the interaction of an armed T_EC_ cell with a target cell displaying specific antigen. They neither display memory marker (CD45RO) nor the homing receptor (CD62L) (7, 8).

Central memory T-cells produce IL-2, whereas T_EM_ cells are characterized by increased secretion of effector cytokines such as IFN-γ and IL-4 (7). T_CM_ cells are moderately long-lived memory cells, capable of differentiating into shorter-lived T_EM_ cells upon antigen stimulation. In turn, T_EM_ cells differentiate into T_EC_ cells. T_EC_ cells represent terminally differentiated T_EM_ cells. Apoptotic death is the outcome of T_EC_ cells upon increased proliferation and antigen exposure (9, 10). A N_TC_ is a mature differentiated T-cell that has not encountered its cognate antigen within the periphery. N_TC_s are usually characterized by the surface expression of L-selectin (CD62L). They do not express memory markers (CD45RO) or activation markers such as CD25, CD44, and CD69. N_TC_s do not proliferate until they encounter their corresponding antigens. Stem memory T-cells have recently been described as sets of memory T-cells in mice and humans comprise 2–4% of CD4^+^ and CD8^+^ T-cells population in the periphery. It is speculated that these cells represent the earliest and long-lasting developmental stage of memory T-cells, displaying stem cell-like properties and expressing a gene profile between naïve and T_CM_ cells (10).

Few studies have intended to identify the memory T cell subsets in leprosy. One earlier study has shown that in fresh and unstimulated blood leukocytes from leprosy patients, memory T cells predominated in the PB form of the disease and correlated with IFN-γ production but such result was not observed in MB patients (11). However, the study did not use an experimental design that allowed classification of memory T cell into subsets.

The correlation between T_CM_ cell expression and pro-inflammatory cytokine production with clinical presentation of multibacillary leprosy relapse case has been investigated (12). Increased frequency of T_CM_ cells in relapsed patients was strongly correlated with the bacillary index and the number of skin lesions was reported by these authors. The study did not give attention to the memory subsets in leprosy spectrum rather they focused on relapse cases. Recently, a cross-sectional study of memory T-cells among type-1 reactions (T1R) has shown that circulating CD4^+^ T_EM_, CD8^+^ T_Ec_, and pro-inflammatory cytokines increased at the onset of T1R in BL patients (13).

Memory T-cells have not been well characterized across leprosy spectrum as well as in leprosy reactions particularly in ENL reactions. In the present study, for the first time we described memory-T cell subsets in LL and ENL reactions before and after prednisolone treatment.

## Materials and Methods

### Study Design

A case–control study with follow-up for 28 weeks was used to recruit 35 patients with ENL reaction and 25 non-reactional LL patient controls between December 2014 and January 2016 at ALERT Hospital, Ethiopia.

### Clinical Case Definitions

The clinical assessment of the patient was used as main diagnostic criteria for ENL cases LL patient controls (4).

#### Erythema Nodosum Leprosum

Erythema nodosum leprosum was clinically diagnosed when a patient had painful tender subcutaneous erythematous skin lesions with or without systemic features such as fever, neuritis, and bone pain occurring in patients with LL or borderline LL.

#### Lepromatous Leprosy

Lepromatous leprosy was clinically diagnosed when a patient had widely disseminated nodular lesions with ill-defined borders and BI above 2.

#### Acute ENL

Acute ENL was defined as an ENL episode lasting less than 24 weeks of prednisolone treatment.

#### Chronic ENL

Chronic ENL was defined as an ENL occurring for 24 weeks or more during which a patient has required ENL treatment either continuously or where any treatment free period has been 27 days or less.

#### Recurrent ENL

Recurrent ENL was defined as a second or subsequent episode of ENL occurring 28 days or more after stopping or steady decrease of steroid treatment for ENL.

#### ENL Recurrence or Flare-up

Erythema nodosum leprosum recurrence or flare-up was defined as the appearance of new ENL nodules after initial control, either while on treatment or after 28 days off treatment.

#### New ENL Case

New ENL case was defined as the occurrence of ENL for the first time in a patient with LL.

### Demographic and Clinical Data Collection

Structured questionnaire were used for clinical data recording for each participant. The ENL International STudy (ENLIST) format was modified and used for clinical data recording. The data collection sheet included the demographic, clinical, and diagnostic information set following the standard guideline at each time point. The clinical information included core points such as the clinical feature, skin lesion, nerve functions, and systemic involvement.

### Clinical Sample Collection

Blood samples were obtained from each patient three times: at recruitment before prednisolone administration and after 12 and 24 weeks of prednisolone treatment foe ENL cases. The 12th week was chosen since the steady decrease in prednisolone after 12th week reaches less than half of the start dose and after 24th-week prednisolone administration normally off unless the patient experiences a chronic condition. The third time-point sample (24th week) was obtained when an ENL patient completed prednisolone treatment and the free treatment period is 15 days or more.

### Peripheral Blood Mononuclear Cell (PBMC) Isolation, Freezing, and Thawing

Ten milliliters of venous blood was collected in sterile BD heparinized vacutainer^®^ tubes (BD, Franklin, Lakes, NJ, USA). PBMCs were separated by density gradient centrifugation at 800 × *g* for 25 min on Ficoll-Hypaque (Histopaque, Sigma Aldrich, UK) as described earlier (14). Cells were washed three times in sterile phosphate-buffered saline solution (1× PBS, Sigma Aldrich^®^, UK) and resuspended with 1 ml of Roswell Park Memorial Institute [RPMI medium 1640 (1×) + GlutaMAX™ + Pen-Strip (GIBCO™, Life technologies™, UK)]. Cell viability was determined by 0.4% sterile Trypan Blue solution (Sigma Aldrich^®^, UK) ranged from 94 to 98%. PBMC freezing was performed using a freezing medium composed of 20% fetal bovine serum (FBS, heat inactivated, endotoxin tested ≤ 5 EU/ml, GIBCO^®^ Life technologies, UK), 20% dimethyl sulfoxide in RPMI medium 1640 (1×). Cells were kept at 80°C for 48–72 h and transferred to liquid nitrogen. Thomson et al. method was used for cell thawing (15). The procedure is briefly described as: cells were removed from liquid nitrogen and taken to a water bath (preadjusted to 37°C) for 30 s until thawed half way and resuspended in 10% FBS in RPMI medium 1640 (1×) at 37°C containing 1/10,000 benzonase until completely thawed, washed two times (5 min each) and counted with trypan blue. A percentage viability of above 90% was obtained. Cell concentration was adjusted to 10^6^ cells/ml in RPMI. Then, 1 ml/well cell suspension was pipetted on 24-well polystyrene cell culture plate (Corning^®^ Costar^®^ cell culture plates) and incubated at 37°C in a 5% carbon dioxide incubator. After an overnight resting, cells were brought to flow cytometry staining room for staining with fluorochromes conjugated antibodies as described below.

### Surface Staining for Flow Cytometry

About 1 × 10^6^/ml cells’ suspension was transferred to round-bottomed FACS tubes (Falcon^®^, BD, UK) followed by washing twice at 400 × g for 5 min at RT. Then, cells were resuspended in 50 µl of PBS and incubated in 1 ml of 10% human AB serum (Sigma Aldrich^®^, UK) for 10 min in the dark at room temperature to block non-specific Fc-mediated interactions and followed by centrifugation at 400 × *g* for 5 min. After resuspending cells in 50 µl PBS buffer, live/dead staining was performed at a concentration of 1 µl/1 ml live/dead stain (V500 Aqua, Invitrogen, Life technologies, UK) for 15 min at 4°C in the dark. Cells were washed once and stained for surface markers directed against anti-human CD3 (APC 450), anti-human CD4 (eFluoro780), anti-human CD8 (PerCp-Cy5.5), anti-human CD62L (APC), and anti-human CD45RO (PE), all from BD, Biosciences, UK. We used for each maker FMO, compensation controls, and unstained cells. Unstained cells were used to exclude the autofluorescence of cells. Cell viability was also checked before staining using 0.4% trypan blue.

### Sample Acquisition and Gating Strategy

After the voltages on the photomultiplier tubes and compensation controls setting, the worksheet area was switched from the normal worksheet to the global worksheet. For inspection purpose, the plots were produced on worksheet such as FSC-H versus FSC-A (to inspect the singlets), FSC-A versus viability marker (to inspect viable cells), and SSC-A versus FSC-A (to inspect populations such as lymphocytes, monocytes, granulocytes, etc.). The threshold for FSC was set to 5,000. For each sample, 500,000–1,000,000 cells were acquired.

**Figure 1:**
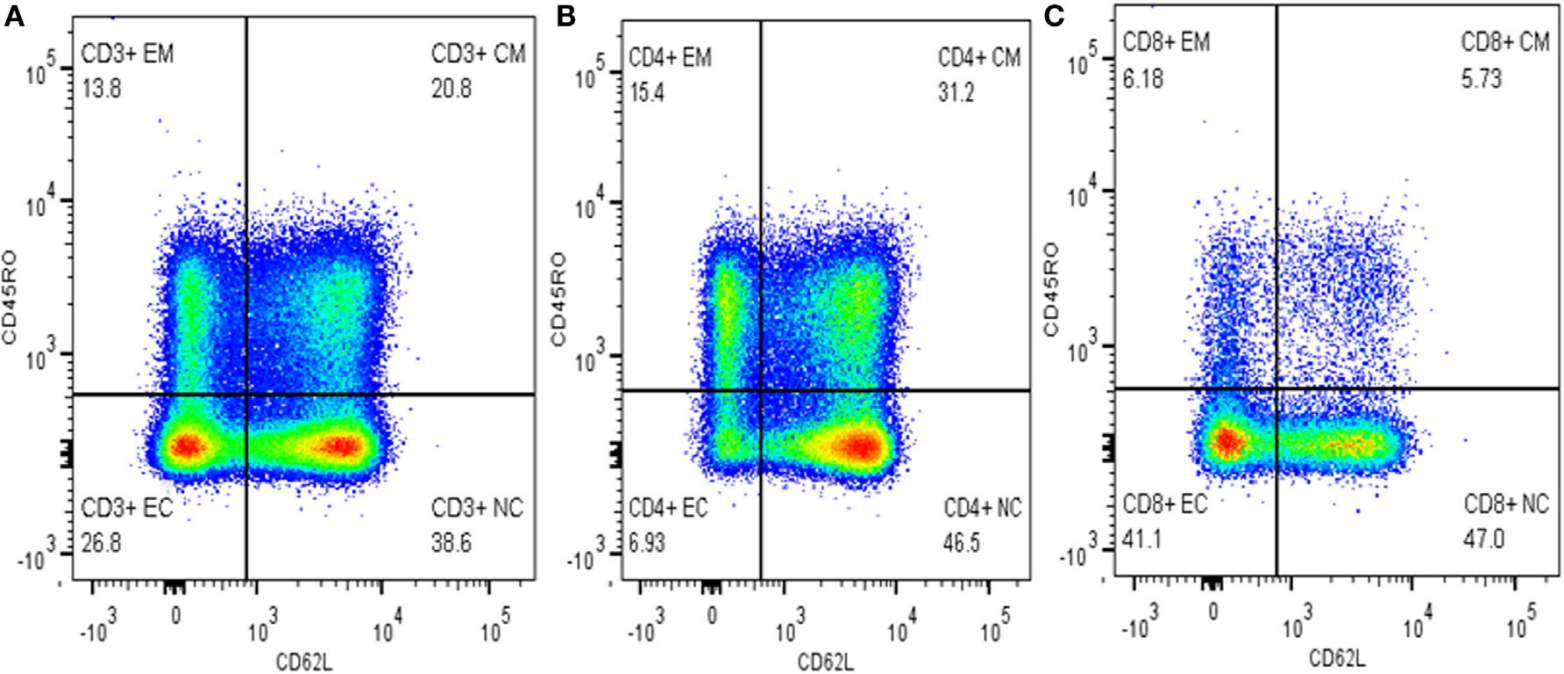
Gating memory T-cells on different cell types. **(A)** Gated on CD3^+^ T-cells, **(B)** gated on CD4^+^ T-cells, and **(C)** gated on CD8^+^ T-cells.

Cells were gated into subpopulations with Flow Jo version 10 (Tree Star, USA) by logicle (bi-exponential) method as recommended by Mohan et al. (16) and Ehlers (17). Activated and memory T-cells were defined as CD3^+^CD62L^−^ and CD3^+^CD45RO^+^, respectively. Memory T-cells were further grouped into T_CM_ cells (CD3^+^CD45RO^+^CD62L^+)^ and activated memory T-cells (CD3^+^CD45RO^+^CD62L^−^). T_EC_ and N_TC_s were defined as CD3^+^CD45RO^−^CD62L^−^ and CD3^+^CD45RO^−^CD62L^+^, respectively (Figure 1). The relative percentage of each subpopulation was copied to excel for each sample and finally an excel spreadsheet electronic data were generated and used for subsequent statistical analysis.

### Statistical Analysis

Differences in percentage of T-cell subsets were computed and statistically tested with two-tailed Mann–Whitney *U* test or Wilcoxon signed rank test for non-parametric distribution using STATA 14 ver. 2 (San Diego, CA, USA). Graphs were produced by GraphPad Prism version 5.01 for Windows (GraphPad Software, San Diego, CA, USA). Median and Hodges–Lehmann estimator were used for result presentation. Hodges–Lehmann is used to measure the effect size for non-parametric data (18). *P*-Value correction was applied for multiple comparisons. A statistical significance level of 0.05 was used to test the difference between cases and controls.

## Results

We described the median percentage of activated T-cells (CD62L^−^), total memory T-cells (CD45RO^+^), and the subgroups of memory T-cells before, during, and after treatment of ENL patients and compared the results with non-reactional LL patient controls as well as within ENL patients.

### Increased T-Cell Activation in Untreated ENL Patients

**Figure 2:**
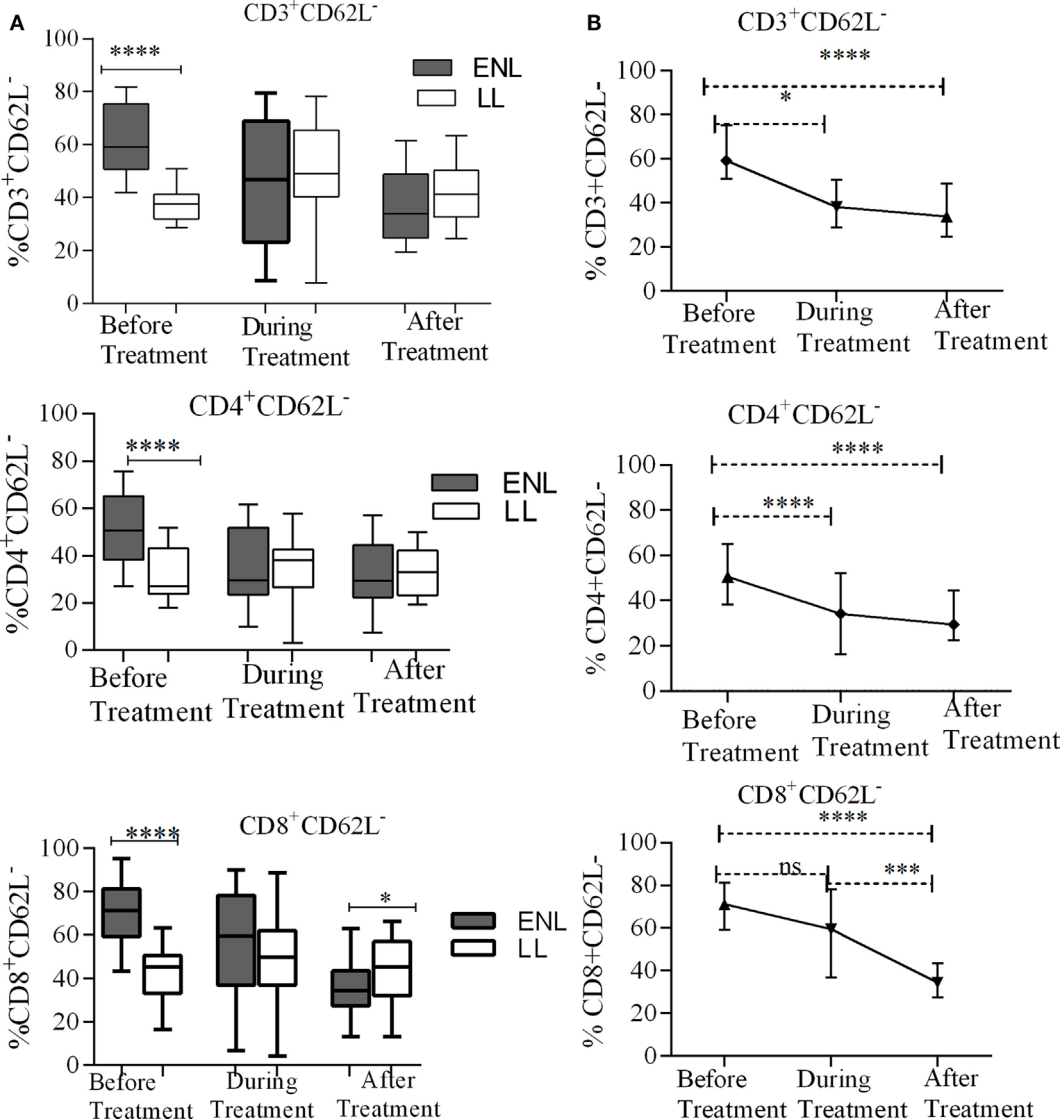
Median percentage of total CD3^+^, CD4^+^, and CD8^+^ activated T-cells: **(A)** in patients with erythema nodosum leprosum (ENL) and lepromatous leprosy (LL) controls before, during, and after treatment, **(B)** within ENL patients before, during, and after treatment. *P ≤ 0.05; ***P < 0.001; ****P < 0.0001. Box and whiskers **(A)** and error bars **(B)** show median ± interquartile range.

The percentage of activated CD3^+^ T-cells (CD3^+^CD62L^−^) was significantly increased in untreated ENL patients (59.3%) in contrast to non-reactional LL patient controls (37.7%) (*P* < 0.0001; ΔHL = 22.4%). However, after treatment statistically a significant difference was not obtained between the groups. Similarly, the median percentage of activated CD4^+^ T-cells (CD4^+^CD62L^−^) was significantly higher (50.7%) in PBMCs of patients with ENL than in LL patient controls (27.1%) before treatment (*P* < 0.0001; ΔHL = 19.1%). However, a significant difference was not observed during and after treatment (*P* > 0.05). Nearly two-third of CD8^+^ T-cells (71.2%) was activated (CD8^+^CD62L^−^) in untreated ENL patients while it was only 45.4% in LL patient controls (*P* < 0.0001; ΔHL = 27.9%). On the other hand, after treatment, the frequency of activated CD8^+^ T-cells was significantly decreased to 34.5% in patients with ENL compared to 45.2% in LL patient controls (*P* ≤ 0.05; ΔHL = 10.1%) (Figure 2A).

When the trend of T-cell activation within ENL is compared before and after treatment, it was found that the median percentage of CD3^+^-activated T-cells before starting prednisolone treatment was higher (59.2%) than during treatment (47.0%) (*P* ≤ 0.05). After treatment, the median percentage of activated CD3^+^ T-cells was decreased to 33.9%, which was significantly lower than before treatment (*P* < 0.0001; ΔHL = 25.5%). Likewise, the median percentage of CD4^+^-activated T-cells was significantly higher (50.7%) before treatment than during treatment (29.7%) (*P* < 0.0001; ΔHL = 17.75%). Similarly, the median percentage of activated CD8^+^ T-cells was significantly higher (71.2%) before treatment than during (59.5%) and after (34.5%) treatment (*P* ≤ 0.05) (Figure 2B).

### Increased Total Memory T-Cells in Untreated ENL Patients

**Figure 3:**
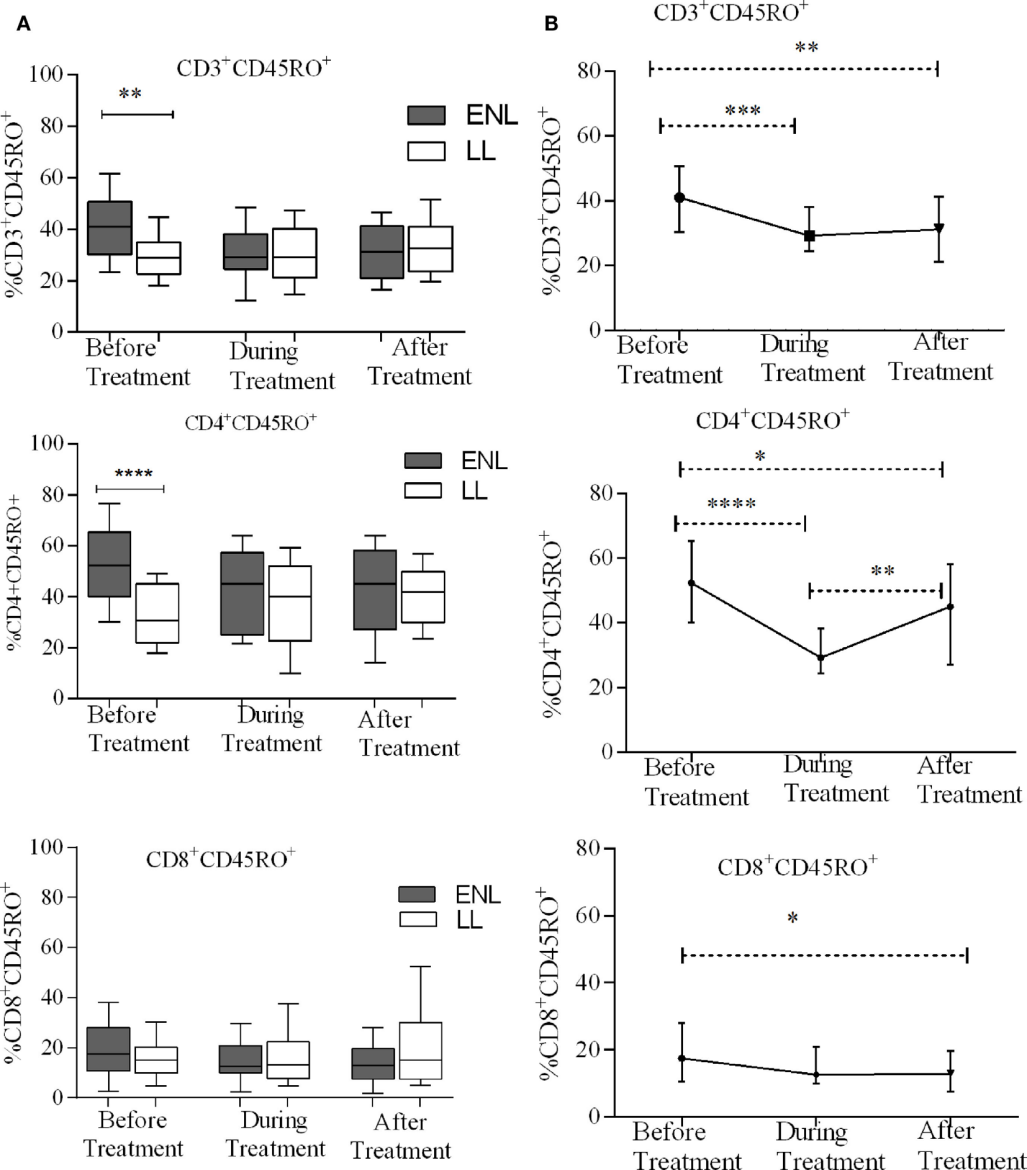
Median percentage of total CD3^+^, CD4^+^, and CD8^+^ memory T-cells: **(A)** in patients with erythema nodosum leprosum (ENL) and lepromatous leprosy (LL) controls before, during, and after treatment, **(B)** within ENL patients before, during, and after treatment. *P ≤ 0.05; **P < 0.005; ***P < 0.001; and ****P < 0.0001. Box and whiskers **(A)** and error bars **(B)** show median ± interquartile range.

Erythema nodosum leprosum patients had significantly increased CD3^+^ total memory T-cell (CD3^+^CD45RO^+^) (40%) compared to LL patient’ controls (28%) before treatment (*P* ≤ 0.005; ΔHL = 10.5%). After treatment, the median percentage of CD3^+^ total memory T-cells in ENL patients and LL controls were 31.2 and 32.7%, respectively, and the result was not statistically significantly different (*P* > 0.05). Similarly, the median percentage of CD4^+^ total memory T-cells (CD4^+^ CD45RO^+^) was increased in untreated ENL patients (50%) compared to LL patient controls (30.5%) (*P* < 0.0001; ΔHL = 20.3%). After treatment, the median percentage of CD4^+^ total memory T-cells in ENL patients (45.0%) was not significantly different compared to LL patient controls (41.8%) (*P* > 0.05). Interestingly, the median percentage of CD8^+^ memory T-cells (CD8^+^CD45RO^+^) was not statistically significantly different between the two groups before and after treatment (Figure 3A).

When the median percentage of total memory T-cells was compared within ENL patients before, during, and after treatment, it was found that the median percentage of CD3^+^ total memory T-cells was significantly higher (41.1%) before treatment than during treatment (29.2%) (*P* ≤ 0.001; ΔHL = 11.2%) and after treatment (31.5%) (*P* ≤ 0.001; ΔHL = 9.8%). Similarly, the median percentage of CD4^+^ total memory T-cells was 52.3% before treatment and significantly decreased to 29.2% during treatment (*P* < 0.0001; ΔHL = 22.1%). On the other hand, unlike CD3^+^ and CD4^+^ memory T-cells, the median percentage of CD8^+^ memory T-cells did not change within ENL patients before, during, and after treatment (Figure 3B).

### Increased T_EM_ Cells in Patients with ENL

Effector memory T-cells are memory T-cells which have lost their CD62L expression while migrating to the tissue and progressively gain functionality with further differentiation to T_EC_ cells also called terminally differentiated T-cells (19). Measurement of T_EM_ cells is the most commonly used method to determine the extent of T-cell activation in a disease state. We measured the proportion of T_EC_ cells in unstimulated PBMCs from patients with ENL and LL controls before, during, and after treatment to prove our hypothesis that ENL is associated with increased T-cell activation.

**Figure 4:**
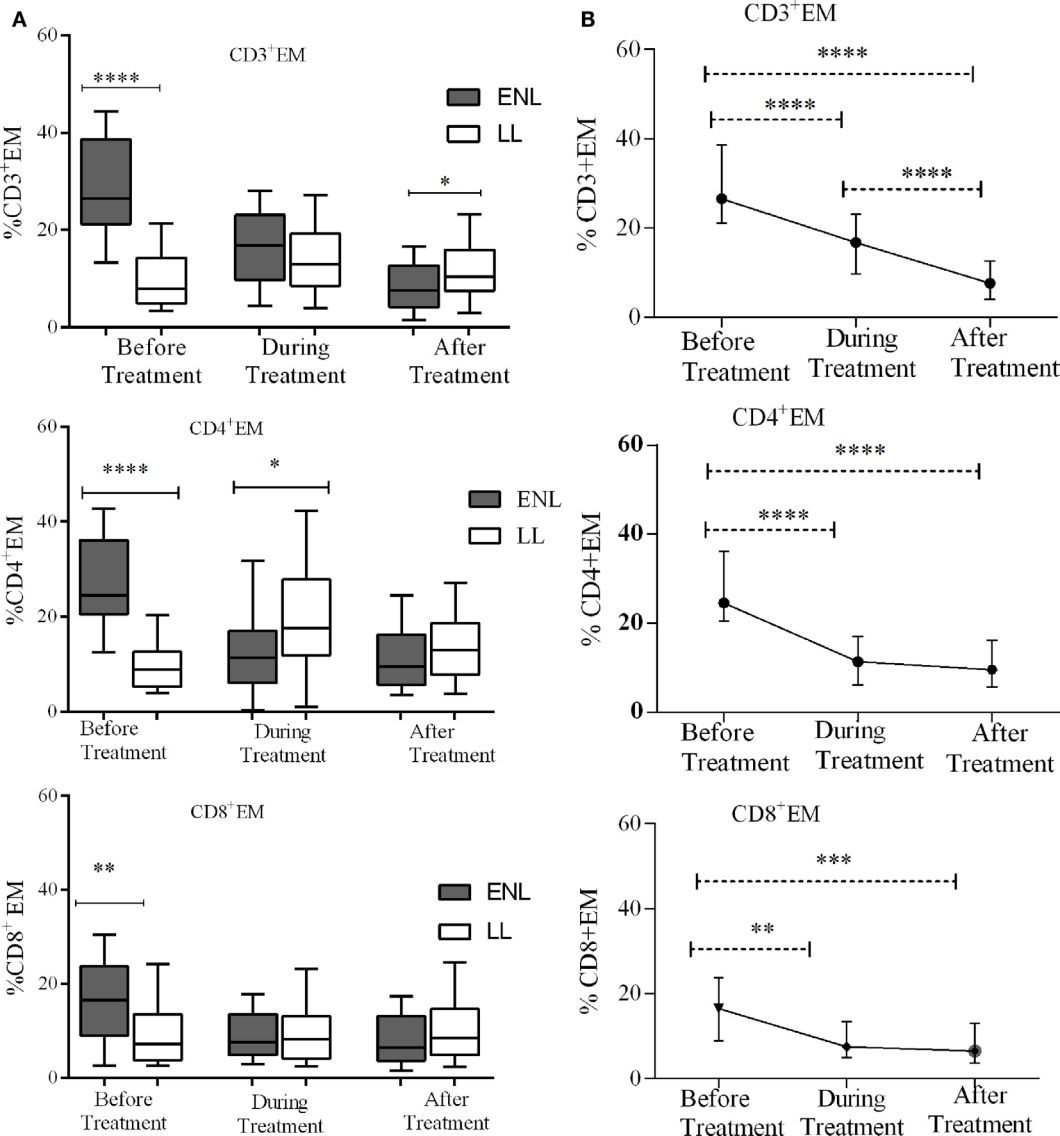
Median percentage of CD3^+^, CD4^+^, and CD8^+^ effector memory T-cells: **(A)** in patients with erythema nodosum leprosum (ENL) and lepromatous leprosy (LL) controls before, during and after treatment, **(B)** within ENL patients before, during, and after treatment. *P ≤ 0.05; **P < 0.005; ***P < 0.001; ****P < 0.0001. Box and whiskers **(A)** and error bars **(B)** show median ± interquartile range.

The median percentage of CD3^+^ T_EM_ cells (CD3^+^CD45RO^+^CD62L^−^) in the PBMCs of patients with ENL was 26.6%, which was significantly higher than in LL patient controls (8.0%) before treatment (*P* < 0.0001; ΔHL = 18.3%). Conversely, the percentage of CD3^+^ T_EM_ cell was found to be lower in patients with ENL (7.6%) than in LL patient controls (10.4%) after treatment (*P* ≤ 0.05; ΔHL = 3.5%). Similarly, the median percentage of CD4^+^ T_EM_ cells (CD4^+^CD45RO^+^CD62L^−^) in the PBMCs of patients with ENL was nearly three times (24.6%) higher than in the PBMCs of LL patient controls (8.9%) before treatment (*P* < 0.0001; ΔHL = 18.4%). However, unlike CD3^+^ T_EC_ cells, the median percentage of CD4^+^ T_EC_ cells was not significantly different between the two groups after treatment (*P* > 0.05). Likewise the median percentage of CD8^+^ T_EM_ cells (CD8^+^CD45RO^+^CD62L^−^) was significantly higher in patients with ENL (16.5%) than in LL patient controls (7.2%) before treatment (*P* < 0.001; ΔHL = 6.7%). However, after treatment, the median percentage of CD8^+^ T_EM_ cells in patients with ENL and LL controls did not show significant difference (Figure 4A).

Trend analysis has shown that T_EM_ cells significantly decreased in ENL patients after prednisolone treatment. Untreated patients with ENL reactions had a higher CD3^+^ T_EM_ cells (26.6%) than during treatment (16.8%) (*P* < 0.0001; ΔHL = 11.88%). The percentage of CD3^+^ T-cells expressing T_EM_ cells (CD3^+^CD45RO^+^CD62L^−^) was considerably decreased to 7.6% after treatment, which was substantially lower than the median percentage of CD3^+^ T_EM_ cells before treatment (*P* < 0.0001; ΔHL = 20.0%). Likewise, the median percentage of CD4^+^ T_EM_ (CD4^+^CD45RO^+^CD62L^−^) cells was more than twofold higher (24.6%) before treatment compared to during treatment (11.4%) and the difference was statistically significant (*P* < 0.0001; ΔHL = 15.54%). The percentage of CD4^+^ T_EM_ cells was remarkably reduced to 9.6% after treatment indicating the decreasing tendency of T-cell activation after prednisolone treatment of patients with ENL. About 16.5% of CD8^+^ T-cells expressed T_EM_ cells (CD8^+^CD45RO^+^CD62L^−^) before treatment. During treatment, the median percentage of CD8^+^ T-cells expressing T_EM_ cells was notably decreased to 7.5%, which was significantly low compared to before treatment (*P* ≤ 0.005; ΔHL = 6.61%). However, unlike CD4^+^ and CD3^+^ T_EM_ cells, the proportion of CD8^+^ T_EM_ cells did not show significant change after treatment (6.5%) (Figure 4B).

### T_CM_ Cells Play Less Role in ENL Reaction

**Figure 5:**
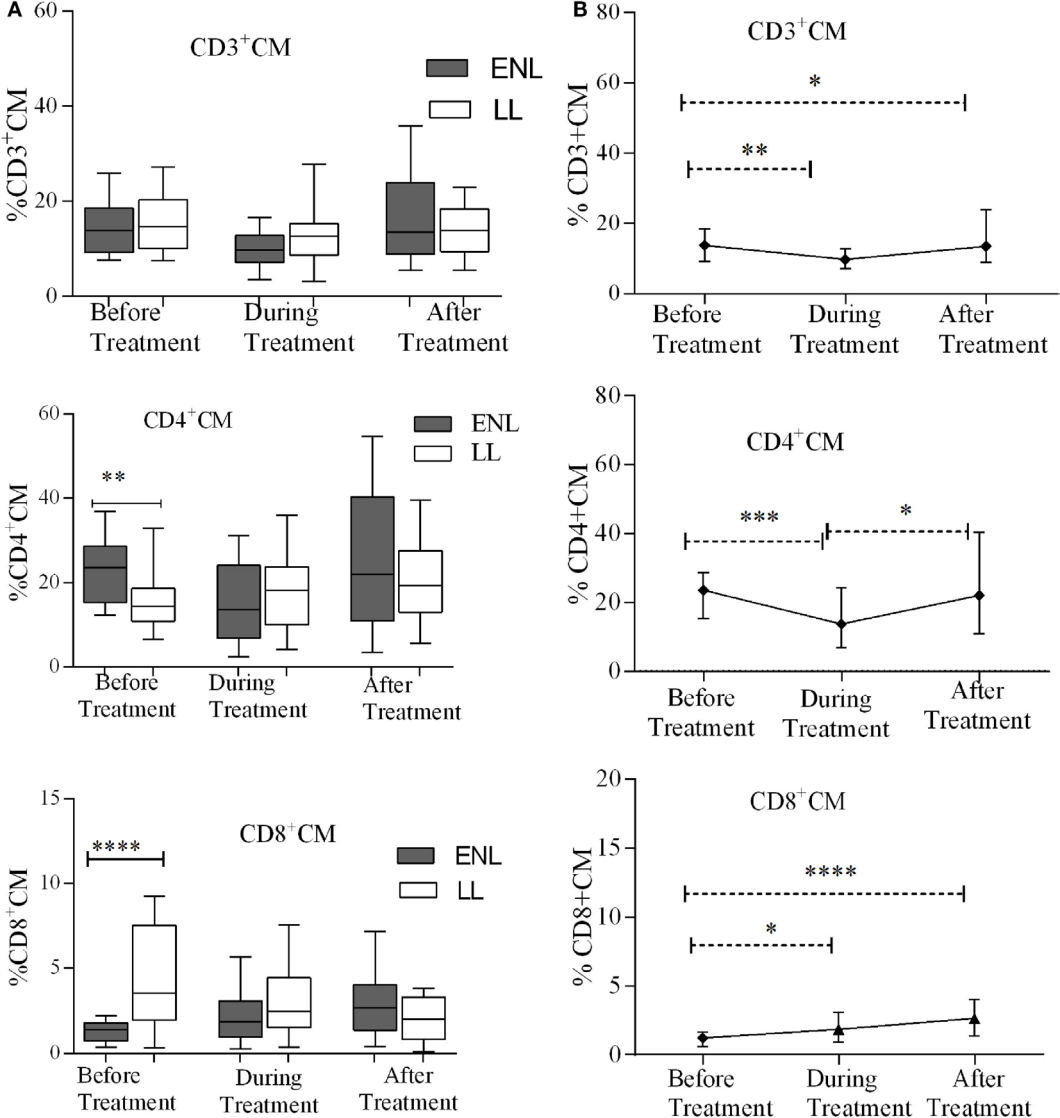
Median percentage of CD3^+^, CD4^+^, and CD8^+^ central memory T-cells: **(A)** in patients with erythema nodosum leprosum (ENL) and lepromatous leprosy (LL) controls before, during, and after treatment, **(B)** within ENL patients before, during, and after treatment. *P ≤ 0.05; **P < 0.005; ***P < 0.001; ****P < 0.0001. Box and whiskers (A) and error bars (B) show median ± interquartile range.

The median percentages of CD3^+^ T_CM_ (CD3^+^CD62L^+^CD45RO^+^) cells in patients with ENL and LL controls before, during, and after treatment were not statistically significantly different (*P* > 0.05). Unlike CD3^+^ T_CM_, the proportion of CD4^+^ T_CM_ (CD4^+^CD62L^+^CD45RO^+^) was significantly higher in patients with ENL (23.5%) than in LL patient controls (14.6%) before treatment (*P* ≤ 0.005; ΔHL = 8.13%). However, the median percentage of CD4^+^ T_CM_ cell was not significantly different between the two groups after treatment. Interestingly, the median percentage of CD8^+^ T_CM_ (CD8^+^CD62L^+^CD45RO^+^) cell was significantly lower in patients with ENL (1.2%) than in LL patient controls (3.5%) before treatment (*P* ≤ 0.0001; ΔHL = 2.3%). After treatment, the percentage of CD8^+^ T_CM_ cell was slightly higher in patients with ENL (2.7%) than in LL patient controls (2.0%); however, the difference was not statistically significant (*P* > 0.05) (Figure 5A).

Comparison within ENL has shown that the percentage of T_CM_ cells was not statistically significantly different before and after treatment (Figure 5B). This confirms that T_CM_ cells do not play significant role in ENL reaction unlike T_EM_ cells.

### Increased T_EC_ Cells in Untreated ENL Patients

**Figure 6:**
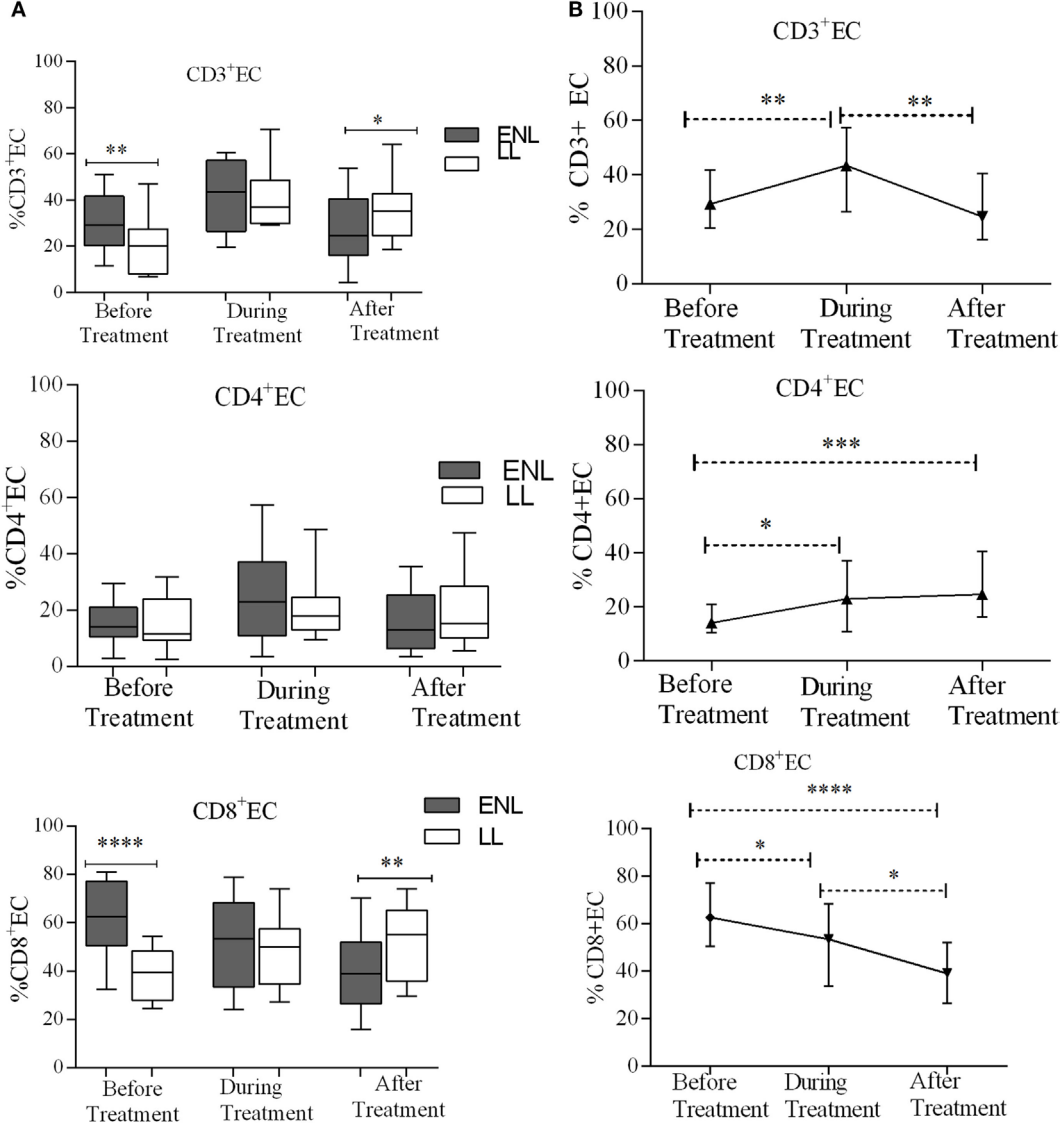
Median percentage of CD3^+^, CD4^+^, and CD8^+^ effector T-cells: **(A)** in patients with erythema nodosum leprosum (ENL) and lepromatous leprosy (LL) controls before, during, and after treatment, **(B)** within ENL patients before, during, and after treatment. *P ≤ 0.05; **P < 0.005; ***P < 0.001; ****P < 0.0001. Box and whiskers **(A)** and error bars **(B)** show median ± interquartile range.

Effector memory T-cells (T_EC_) are short-lived unlike memory T-cells and they shortly undergo apoptosis once they meet their cognate antigens. Nearly one-third (29.3%) of CD3^+^ T-cells were effector cells (T_EC_) in the PBMCs of patients with ENL with the corresponding value of 20.0% in LL patient controls before treatment and the difference was statistically significant (*P* ≤ 0.001; ΔHL = 9.0%). After treatment, the median percentage of CD3^+^ T_EC_ cell was significantly decreased in patients with ENL (24.6%) compared to LL patient controls (35.3%) (*P* ≤ 0.05; ΔHL = 8.6%). With regard to the median percentage of CD4^+^ T_EC_ cell, a statistically significant difference was not obtained between the two groups before, during, or after treatment. The median percentage of T_EC_ cell expression in CD8^+^ T-cells in patients with ENL was 62.7%, which was considerably higher than the value obtained for LL patient controls (39.5%) before treatment (*P* < 0.0001; ΔHL = 25.8%). Similar to CD3^+^ T_EC_ cell, the proportion of CD8^+^ T_EC_ cell in patients with ENL was significantly decreased (38.9%) compared to LL patient controls (55.2%) after treatment (*P* ≤ 0.005; ΔHL = 14.4) (Figure 6A).

The median percentage of CD3^+^ T_EC_ cells was lower before treatment (29.3%) than during treatment (43.4%) (*P* ≤ 0.005). After treatment, the percentage of these cells decreased to 24.6%. Similarly, the median percentage of CD4^+^ T_EC_ cells was lower before treatment (14.0%) than during treatment (22.9%) (*P* ≤ 0.05). After treatment, the percentage of CD4^+^ T_EC_ cells was decreased by half (12.1%) than during treatment (*P* ≤ 0.05). Like CD3^+^ T_EC_ cells, the percentage of CD4^+^ T_EC_ cells did not show significant difference before and after treatment. On the other hand, the median percentage of CD8^+^ T_EC_ cells was higher (62.7%) before treatment than during treatment (53.4%) (*P* ≤ 0.05). After treatment, the percentage of CD8^+^ T_EC_ cells was considerably decreased to 38.9% compared to during treatment (*P* ≤ 0.05) and before treatment (*P* < 0.0001) (Figure 6B).

### N_TC_s Decreased in ENL Patients Compared to Non-Reactional LL Controls

**Figure 7:**
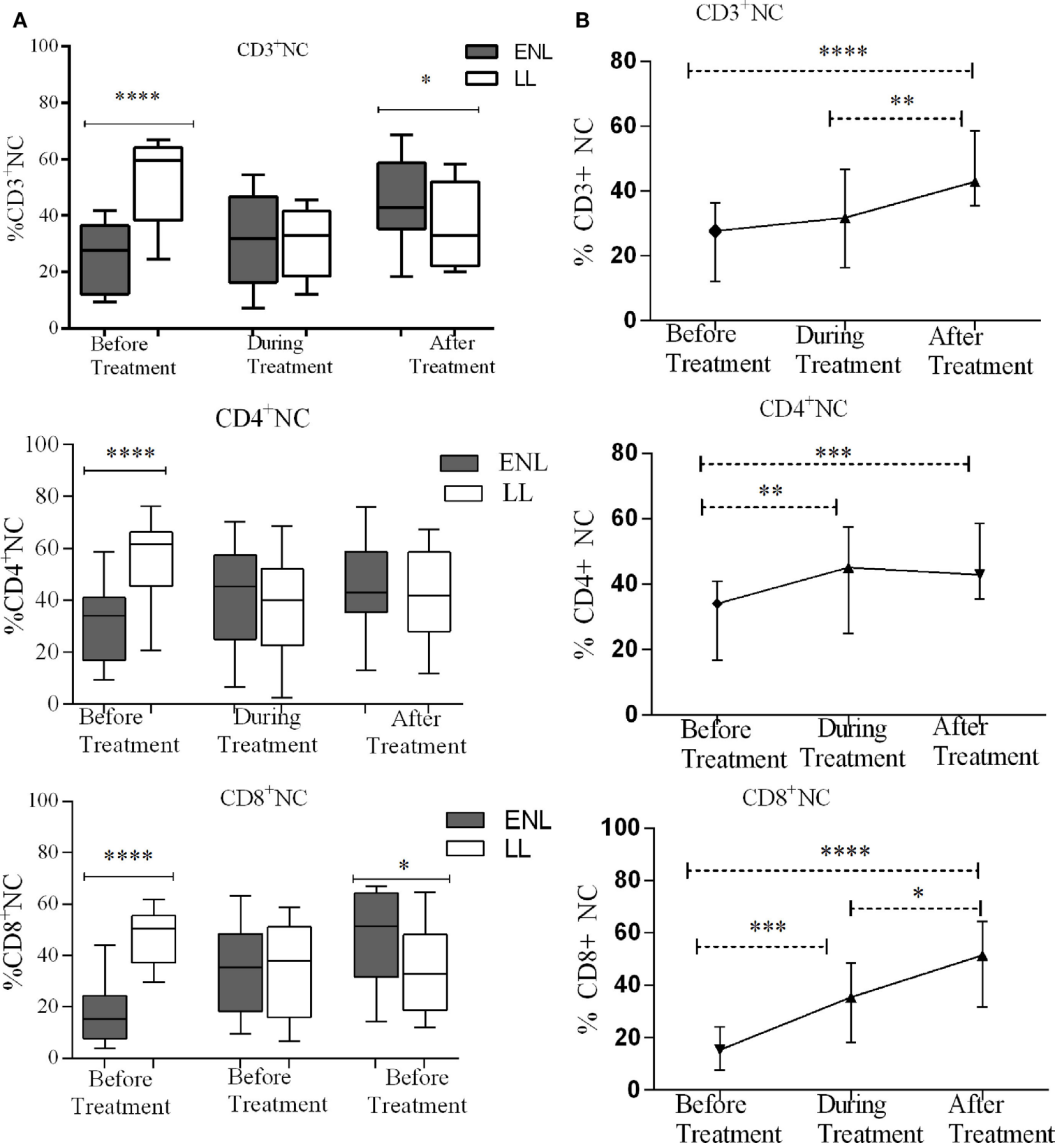
Median percentage of CD3^+^, CD4^+^, and CD8^+^ naïve T-cells: **(A)** in patients with erythema nodosum leprosum (ENL) and lepromatous leprosy (LL) controls before, during, and after treatment, **(B)** within ENL patients before, during, and after treatment. *P ≤ 0.05; **P < 0.005; ***P < 0.001; ****P < 0.0001. Box and whiskers **(A)** and error bars **(B)** show median ± interquartile range.

Despite the higher bacterial load in patients with LL patients, the median percentage of CD3^+^ N_TC_s was significantly higher (59.5%) in these patients compared to that in patients with ENL (27.7%) before treatment (*P* < 0.0001; ΔHL = 26.5%). During treatment, the median percentage of CD3^+^ N_TC_s significantly decreased to 32.9% in LL patient controls while in patients with ENL the percentage was slightly increased to 31.8%. After treatment, the percentage of CD3^+^ N_TC_s was further increased to 42.9% in patients with ENL but did not change in LL patient controls (33.0%), and the difference between the two groups was statistically significant. Similarly, the median percentage of CD4^+^ N_TC_s in patients with ENL (34.0%) was significantly lower than that in LL patient controls (61.5%) before treatment (*P* < 0.0001; ΔHL = 25.6%). However, during and after treatment the median percentage of CD4^+^ N_TC_s did not show statistically significant difference (*P* > 0.05). On the other hand, the median percentage of CD8^+^ N_TC_s in patients with ENL was more than three times lower (15.4%) than in LL patient controls (50.5%) before treatment (*P* < 0.0001; ΔHL = 31.6%). During treatment, while the median percentage of CD8^+^ N_TC_s increased to 35.5% in patients with ENL, it was decreased to 38% in LL patient controls. After treatment, the median percentage of CD8^+^ N_TC_s in patients with ENL and LL controls was 51.5 and 32.8%, respectively, and the difference between the two groups was statistically significant (*P* ≤ 0.05; ΔHL = 14.4%) (Figure 7A).

Comparison within ENL has shown that the percentage of CD3^+^ T_NC_ cell was lower (27.7%) before than after treatment (42.9%) (*P* < 0.0001). Similarly, the percentage of CD4^+^ T_NC_ cells was lower (34.1%) before treatment than after treatment (40.4%) (*P* ≤ 0.005). The percentage of CD8^+^ T_NC_ cells was 15.4% before treatment but increased to 51.5% after treatment and it was significantly higher than before treatment (*P* < 0.0001) (Figure 7B).

### Decreased Regulatory T-Cells (T_reg_)/T_EM_ Cells Ratio in ENL Patients Compared to LL Controls

**Figure 8:**
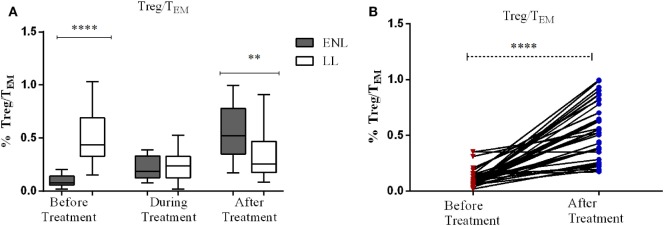
The median percentage of regulatory T-cells (T_reg_)/effector memory T-cells (T_EM_) cells: **(A)** in patients with erythema nodosum leprosum (ENL) and lepromatous leprosy (LL) controls before, during, and after treatment, **(B)** within ENL patients before, during, and after treatment. **P < 0.005; ***P < 0.001; ****P < 0.0001.

The median percentage ratio of T_reg_ (CD3^+^CD4^+^CD25^+^FoxP3^+^) to T_EM_ (T_reg_/T_EM_ cells) was significantly lower in untreated patients with ENL (0.077) than in LL controls (0.44) before treatment (*P* ≤ 0.0001). However, after treatment the median percentage ratio of T_reg_/T_EM_ cells was significantly increased in patients with ENL (0.522) compared LL controls (0.255) (*P* ≤ 0.005) (Figure 8A).

When the ratio of T_reg_/T_EM_ cells is compared within ENL groups, it was found that patients with ENL had a significantly lower median percentage of T_reg_/T_EM_ cells (0.077) before treatment than after treatment (0.552) (*P* < 0.0001) (Figure 8B).

## Discussion

The ability of inflammatory cells to respond to pathogens is crucial for maintaining healthy conditions. In mammals, lymphocytes leave the circulation and migrate to secondary lymphoid organs, such as lymph nodes, where antigens are presented. When an antigen is encountered, directed release of immune cells to sites of inflammation orchestrates host defense. Both constitutive and inflammatory leukocyte trafficking is controlled by adhesion molecules. The initial tethering of leukocytes to the endothelium and to other leukocytes is assisted by selectins, particularly by L-selectins. L-selectin directs neutrophils and lymphocytes to sites of inflammation. Upon T-cell activation L-selectin is shed from the leukocyte surface (8, 20).

In this study, the status of T-cell activation and the different subtypes of memory T-cells were investigated. In leprosy, the different classes of memory T-cells have not been studied. To our knowledge, it is for the first time that the different subtypes of memory T-cells and T-cell activation are phenotypically described in patients with ENL and LL. We are the first to study the status of T-cell activation in patients with ENL. Not only T-cell activation and the different classes of memory T-cells are described but also the changes of these immune cells over time before, during, and after prednisolone treatment were investigated. Therefore, our present data will provide basic information for future studies involving T-cell activation and memory T-cells in ENL.

### Increased Activation of T-Cells in Entreated ENL

In our study, patients with ENL had significantly higher percentage of CD3^+^, CD4^+^, and CD8^+^ activated T-cells than LL patient controls before treatment. However, after prednisolone treatment, T-cell activation was not significantly different in patients with ENL and LL controls except for the transient activation of CD8^+^ T-cells. This result is the evidence of T-cell activation in patients with ENL reactions. *In vitro* stimulation of PBMCs from patients with ENL reaction with *M. leprae* whole-cell sonicate has shown an increased T-cell response as assessed by IFN-γ and TNF-α production (data not shown). Excessive T-cell activation as a cause of tissue damage in several inflammatory diseases has been described in many studies (21, 22). The finding of T-cell activation in patients with ENL implies the involvement of T-cell activation in the pathogenesis of ENL.

In this study, changes in the percentage of activated T-cells were investigated before, during, and after prednisolone treatment within ENL groups. The percentages of CD3^+^, CD4^+^, and CD8^+^ activated T-cells were significantly reduced during and after prednisolone treatment. The reduction of activated T-cells following prednisolone treatment may be explained by the immunosuppressive activity of prednisolone. Although studies showing the effect of prednisolone treatment on T-cell response in leprosy reactions is lacking, several studies in other inflammatory diseases have shown the suppressive effect of prednisolone on the T-cell response (23–25). Consequently, our present findings provide evidence that the effect of prednisolone treatment of patients with ENL could be through suppressing T-cell responses.

### T_EM_ Cells Significantly Increased in Untreated ENL Patients

**Figure 9:**
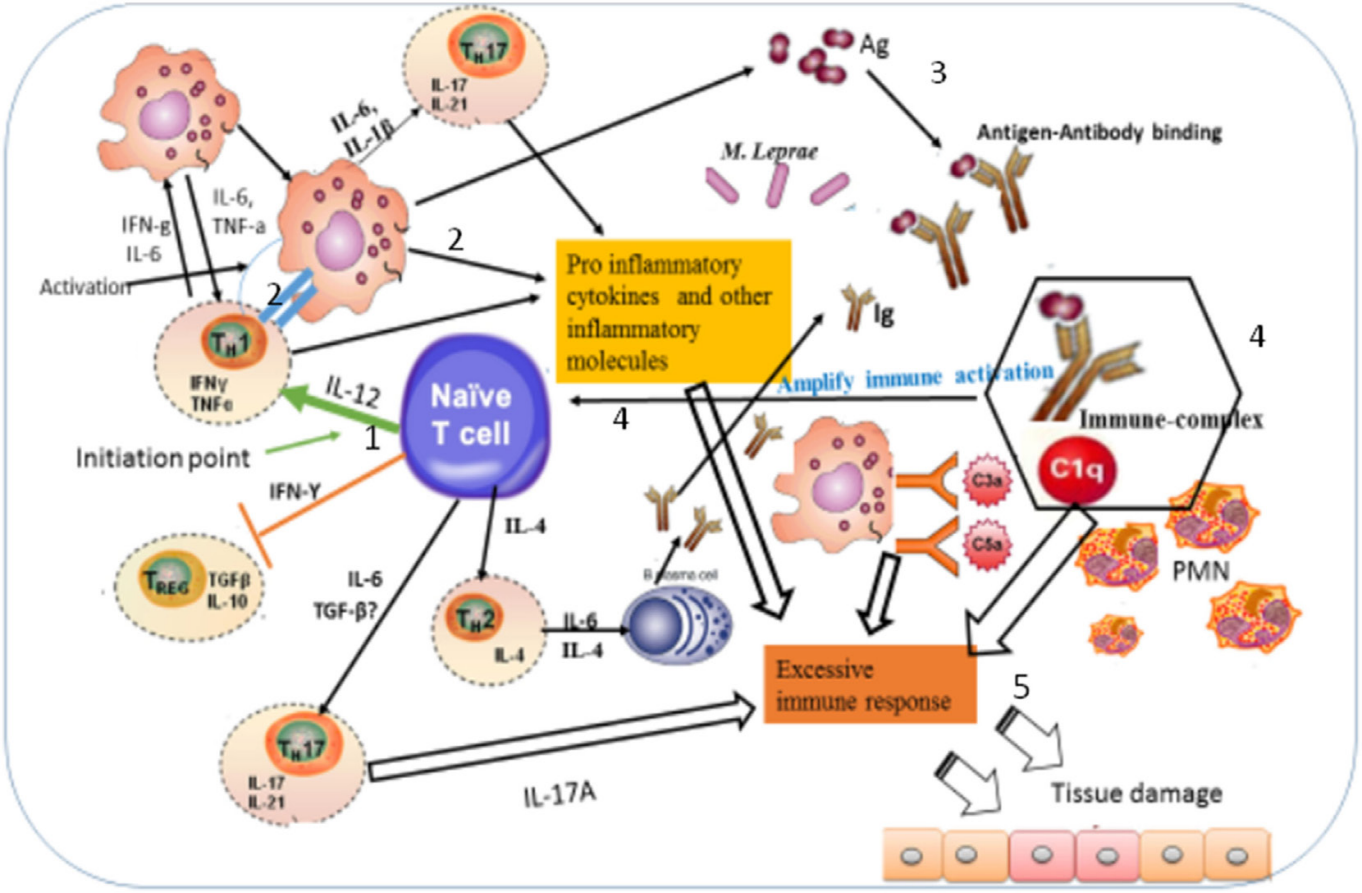
Illustration of the possible immunopathogenesis of erythema nodosum leprosum (ENL) based on the present findings. Based on the finding of this study, the immunopathogenesis of ENL can be illustrated using the following key steps: (1) spontaneous activation of T-cells leading to activation of macrophages that are already loaded with intact *Mycobacterium leprae*. Then, the activation of macrophages produces three key events: processing *M. leprae* and releasing of the processed antigens; antigen presentation to Th1 and production of pro-inflammatory cytokines such as IFN-γ and TNF-α and other inflammatory molecules. The causes of spontaneous T-cell activation should be investigated in the future. (2) Antigen presentation to Th1 stimulates Th1 to produce chemokines, which recruits macrophages to the site of antigen deposition, pro-inflammatory cytokines (IFN-γ, TNF-α), and other inflammatory mediators that increase expression of vascular adhesion molecules. (3) The processed and released antigens bind to the presynthesized antibody leading to antigen–antibody complex formation, which in turn recruits C1q complement and hence immune-complex formation. Following the immune-complex formation, neutrophils will be recruited to the site of immune-complexes. (4) Once immune-complex is formed, it amplifies the immune response which leads to aggressive antigen presentation, immunoglobulin synthesis, and activation of other inflammatory T-cells. (5) The pro-inflammatory cytokines and other inflammatory molecules released from macrophages, Th17, and Th1 and the immune-complex formation beyond clearance lead to tissue damage as sketched above.

The median percentage of CD3^+^, CD4^+^, and CD8^+^ T-cells expressing T_EM_ cells in the PBMCs of patients with ENL was significantly high compared to LL patient controls before treatment. Such a difference was not observed after prednisolone treatment of patients with ENL. This implies that in patients with ENL, there is a continuous activation of T-cells. This continuous T-cell activation could lead to an excess antibody–antigen complex formation but insufficient to clear bacilli from lesions (26). This means the rate of immune-complex formation is greater than the rate of immune-complex clearance, which leads to further tissue damage through recruitment of inflammatory molecules to the site of immune-complex deposition. In LL, there is high load of bacilli. The macrophages are laden with this intact bacillus but unable to process and present to T-cells for further action. LL patients are also characterized by the presence of high antibody titer although these antibodies play little or no role to protect the multiplication of *M. leprae* in these patients. Spontaneous activation of T-cells could lead to the macrophage activation or B-cell activation or both. Macrophage activation results in the processing of the bacilli and releasing the processed bacterial components, which further activate other immune cells. The activation of B-cells by T-cells could produce functional antibodies, which form immune-complex with the already accumulated bacterial antigens. However, this scenario is less likely to happen as it is confirmed that the different subtypes of B-cells did not significantly different in ENL and LL patients (data not shown). Whichever the activation takes place, if excess immune-complex is formed due to the presence of excess antigens in the body, it leads to more immune-complex formation than immune-complex clearance, and hence, some immune-complexes deposit in tissues and often induce inflammatory responses and can cause tissue damage. The causes of tissue damage could be due to the action of complement cleavages, which induces the release of tissue damaging granules such as histamine or the recruitment of inflammatory cells such as neutrophils and macrophages into the tissue. However, this assumption needs further study to give definitive evidence. Previous studies have suggested that human T_EM_ cells display characteristic sets of chemokine receptors and adhesion molecules that are required for homing to inflamed tissues and they have an immediate effector function (20, 27). This situation could amplify the immune response and hence further aggravate tissue damage in a vicious circle (Figure 9).

A kinetic analysis of the percentage of T_EM_ cells in the PBMCs from patients with ENL before, during, and after prednisolone treatment showed that the median percentage of T_EM_ cells was decreased from 27% before treatment to 8% after treatment with an effect size of 20%. It has been described in previous sections that T_EM_ cells rapidly differentiate to T_EC_ cells upon antigenic stimulation. The consequence of excessive expression of T_EM_ cells is detrimental to tissue damage (9). In apparently healthy individuals, T_EC_ cells do not express CD62L or CCR7 and hence do not home to lymph nodes (10). A breakdown in the compartmentalization of such T_EC_ cells is predicted to have unfavorable consequences for the immune system. Hence, the finding of an increased percentage of T_EM_ cells in patients with ENL before treatment suggests that T_EC_ cells take part in the process of tissue damage observed in these patients.

### More T-Cells in ENL Patients Are Antigen Experienced than in LL Patients

Interestingly, the median percentage of N_TC_s’ expression in CD3^+^, CD4^+^, and CD8^+^ T-cells in the PBMCs of patients with ENL was significantly low compared to LL controls before treatment implying that T-cells from patients with ENL have more antigenic exposure than those from LL. It is important to note that patients with ENL had LL before they developed ENL during which a high percentage of N_TC_s is expected since higher percentage of N_TC_s was investigated in this study in LL patients. Following the development of ENL, the percentage of N_TC_s drops to below 30%. This implies that either those previously N_TC_s became responsive and able to recognize their cognate antigen or the newly produced T-lymphocytes during the onset of ENL reactions are able to recognize and respond to the existing *M. leprae* antigens unlike in LL. It is an established fact that despite the high bacterial load in patients with LL, the cell-mediated immune response is virtually absent (28, 29). In addition to a specific loss of cell-mediated immune response against *M. leprae* in these patients, a relative impairment of the ability of lymphocytes to react *in vitro* has also been reported. Furthermore, lymph nodes from patients with LL show a deficiency of these cells in those areas associated with the development of cell-mediated immune responses (14, 28–31). Therefore, the significantly reduced median percentage of N_TC_s in blood from patients with ENL reaction provides an evidence of T-cell responsiveness in patients with ENL. This means that unlike in LL patients, the N_TC_s in ENL patients are primed in recognition of the *M. leprae* antigen (Figure 9).

The median percentage of N_TC_s was significantly increased after prednisolone treatment within ENL groups. The percentage of N_TC_s in untreated patients with ENL reactions was less than 30% and was increased to nearly 50% after treatment. Previous studies have shown that prednisolone treatment increases in a dose-dependent manner the percentage of N_TC_s in experimental mice. Hence, the finding of high percentages of N_TC_s after prednisolone treatment of patients with ENL may be explained by the fact that prednisolone resolves inflammation at least partly by increasing the percentage of N_TC_s which concurrently reduce the percentage of activated T-cells.

In our present work, we have shown that ENL reaction is associated with increased T-cell activation. Our findings suggest that ENL reaction is a T-cell-mediated pathology. Hence, the T-cell-mediated pathology of ENL will provide further insights into disease mechanisms and will potentially result in promising new therapeutic options. Therefore, future ENL studies should consider these fertile areas to improve the treatment and management of ENL. It might also be important to think of to interfere with T-cell trafficking into tissues and thereby reducing inflammation in these patients.

## Ethics Statement

Informed written consent for blood samples was obtained from patients following approval of the study by the Institutional Ethical Committee of London School of Hygiene and Tropical Medicine, UK (#6391), AHRI/ALERT, Ethiopia (P032/12), and the National Research Ethics Review Committee, Ethiopia (#310/450/06). All data have been analyzed and reported anonymously.

## Author Contributions

EN and DL formulated the study questions. EN, DL, HD, SW, and KB designed the study protocol. EN, BE, and KB conducted the experiment. AA, HD, and DL supervised the study. EN analyzed the data. All the authors contributed to the interpretation of the data. EN drafted the manuscript. KB, SW, BE, RH, AA, HD, and DL revised the manuscript. All the authors read and approved the final version for publication. All the authors agreed to be accountable for all aspects of the work in ensuring that questions related to the accuracy or integrity of any part of the work are appropriately investigated and resolved.

## Conflict of Interest Statement

The authors declare that the research was conducted in the absence of any commercial or financial relationships that could be construed as a potential conflict of interest.
